# The Health-Promoting Effects and the Mechanism of Intermittent Fasting

**DOI:** 10.1155/2023/4038546

**Published:** 2023-03-03

**Authors:** Simin Liu, Min Zeng, Weixi Wan, Ming Huang, Xiang Li, Zixian Xie, Shang Wang, Yu Cai

**Affiliations:** ^1^College of Pharmacy, Hubei University of Chinese Medicine, Wuhan 430065, China; ^2^College of Clinical Chinese Medicine, Hubei University of Chinese Medicine, Wuhan 430065, China

## Abstract

Intermittent fasting (IF) is an eating pattern in which individuals go extended periods with little or no energy intake after consuming regular food in intervening periods. IF has several health-promoting effects. It can effectively reduce weight, fasting insulin levels, and blood glucose levels. It can also increase the antitumor activity of medicines and cause improvement in the case of neurological diseases, such as memory deficit, to achieve enhanced metabolic function and prolonged longevity. Additionally, IF activates several biological pathways to induce autophagy, encourages cell renewal, prevents cancer cells from multiplying and spreading, and delays senescence. However, IF has specific adverse effects and limitations when it comes to people of a particular age and gender. Hence, a more systematic study on the health-promoting effects and safety of IF is needed. This article reviewed the research on the health-promoting effects of IF, providing a theoretical basis, direction for subsequent basic research, and information related to the clinical application of IF.

## 1. Introduction

The World Health Statistics 2020 report showed that 41 million people die from noncommunicable diseases worldwide, contributing to 71% of the world's total deaths, wherein the top four diseases include cerebrovascular disease, cancer, chronic respiratory disease, and diabetes [1]. The increase in diabetes-related mortality is associated with an increased prevalence of obesity. Since 2000, the prevalence of age-specific fertilizer obesity in adults has increased by 1.5 times globally, and the prevalence and overweight rates in children have also increased significantly [1]. The increase in cancer incidence and mortality is related to population aging [2], which also has made diseases like sarcopenia and neurodegenerative diseases common [3, 4].

The purpose of the traditional continuous calorie restriction (CR) diet is to reduce the energy intake to 50%~70% of the target for a long time. Although the weight loss effect is significant, it is not easy to adhere to it. For example, the experiment of Xiao proved the compliance of CR decreased by about 40% compared with that of IF [5]. To ameliorate this situation, intermittent fasting (IF), which is defined as a periodic eating pattern with little or no energy intake for a while following normal eating, is used as an alternative treatment [6].IF includes alternate-day fasting (ADF), modified fasting (MF), time-restricted fasting (TRF), and fasting-mimicking diet (FMD), and the information of the classification table is shown in Table 1. Modern medicine shows that IF has been proved to be effective in alleviating obesity symptoms, reducing the risk of related metabolic diseases and age-related diseases, and improving the health indicators of healthy individuals and patients with chronic diseases. Besides, it plays a variety of health-promoting biological effects based on multiple pathway mechanisms. This study reviews the health-promoting effects and mechanisms of IF, providing guidance for subsequent basic research and clinical application of IF and proposing new ideas for adjuvant treatment of various diseases.

## 2. The Health-Promoting Effects of IF

### 2.1. IF in Obesity

The World Health Organization defines “being overweight” and “obesity” as abnormal or excessive fat accumulation that threatens health. Obesity is a complex multifactor disease. Since 1980, the incidence of being overweight and obese has doubled worldwide, and currently, almost one-third of the world's population falls under these categories [7]. The World Health Statistics 2020 report also showed an uptrend in obesity prevalence. Since 2000, the global adult obesity prevalence increased by 1.5 times, and the incidence of obesity has doubled among children [8].

Several studies showed that IF can improve obese patients' health indicators. Bhutani et al. proved that ADF, combined with appropriate exercise, can effectively reduce obese patients' weight and low-density lipoprotein cholesterol (LDL) levels and improve other indicators [9]. Arat et al. demonstrated that fasting during Ramadan could effectively improve relevant health indicators in healthy adult males [10]. Gur et al. demonstrated that IF during Ramadan reduces visceral fat thickness (VFT) in pregnant women without affecting fetal development and amniotic fluid levels, but there is insufficient evidence to prove the harmlessness of IF in pregnant women [11]. There is also evidence indicating that obesity causes delayed lactation in mothers [12]. Madkour et al. showed that fasting during Ramadan could prevent oxidative stress and adverse metabolic disorders in nondiabetic obese patients [13]. Johnf et al. found that in mice, weight loss can effectively mediate the decrease in blood glucose, leptin, TNF-*α*, and insulin-like growth factor-1 (IGF-1) levels [14]. Deng et al. proved that IF can improve the metabolic disorder of white adipose tissue (WAT) in mice under a high-fat diet [15] (Table 2). Studies have shown that IF leads to a well-sustained energy limit, similar to the traditional weight-loss effect [16]. At the same time, experiments proved that IF increases the vascular endothelial growth factor (VEGF) expression of WAT and in turn induces angiogenesis, the polarization of macrophages in the adipose tissues, and the browning of adipocytes, leading to increased insulin sensitivity and effective reduction in obesity [17].

### 2.2. IF in Type 2 Diabetes

The global incidence of type 2 diabetes mellitus (T2DM) is rising; this will increase to 10.2% by 2030 (578 million) and to 10.9% by 2045 (700 million) [18]. The occurrence of T2DM is related to abnormal inflammatory cytokine and oxidative stress (OS) markers. Studies have shown that weight loss can regulate the biomarkers of inflammatory cytokines and oxidative stress [19]. OS is the imbalance between the body's oxidation and antioxidants. It causes increased inflammatory infiltration, protease secretion, and the production of many oxidative intermediates [20].

The cellular overload of glycogen reduces the uptake of glucose by the tissues, thus increasing glucose concentration. IF promotes glucose uptake by the organ tissues and enhances the ability to store glucose as glycogen [21]. While fasting, the liver maintains blood glucose levels by regulating metabolic pathways, such as increased gluconeogenesis (GNG), as well as by upregulating the activity of two restriction enzymes, namely, phosphoenolpyruvate carboxykinase (PEPCK) and glucose-6- phosphatase (G6Pase), to regenerate glucose and maintain blood glucose levels [22]. Nonshivering thermogenesis can reestablish energy balance, thus counteracting the effects of increased energy intake [23]. Experimental results showed that IF mediates the increase of VEGF level in white fat, and M2 macrophages are activated, leading to the browning of white fat [17]. This subsequently mediates nonshivering thermogenesis (NST) and reduces obesity.

Cognitive function decline is one of the most severe problems of T2DM. Diabetes can induce cognitive dysfunction by inhibiting the PI3K/AKT signaling pathway, inhibiting the activity of cAMP response element-binding protein (CREB), and downregulating the expression of the brain-derived neurotrophic factor (BDNF) [24]. However, studies indicated that IF can remake the intestinal microbiome, improve hippocampal synapse ultrastructure, enhance the hippocampal mitochondrial biogenesis and energy metabolism gene expression, and thus effectively alleviate cognitive and motor disorders [25]. The effect of IF on neurogenesis is achieved by downregulating the IGF-1 and PKA signaling [26].

In a mouse model, IF effectively alleviated diabetic retinopathy (DR) by remodeling the intestinal flora, prolonging survival time, reducing DR endpoints with unchanged glycosylated hemoglobin, reducing the TNF-*α* mRNA levels in the retina, and protecting the retina by activating G protein-coupled bile acid receptors to protect the retina and prevent DR [27].

**Figure 1:**
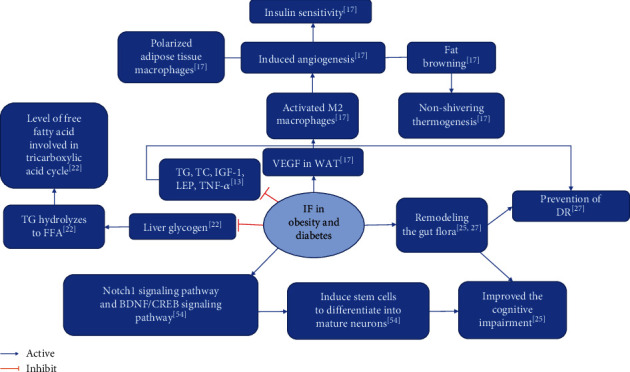
Summary of biological mechanisms of IF intervention in obesity and T2DM.

Experiments by Alharbi et al. demonstrated that Ramadan fasting effectively suppresses weight gain in diabetic patients [28]. Arnason et al. showed that ADF effectively reduces weight and blood glucose levels in diabetic patients [29]. Klempel et al. demonstrated that MF effectively prevents the occurrence [30] (Table 3).  Figure 1 shows the summary diagram of the biological mechanisms of simultaneous IF intervention in the case of obesity and diabetes.

### 2.3. IF in Cancer

Epidemiology showed that by 2020, there was an estimated 19.3 million new cancer cases worldwide, and by 2040, the number of global cancer cases is expected to reach 28.4 million [2]. Studies have also shown that dietary patterns influence the rate and progression of common cancers [31]. Additionally, the anticancer effects of IF have been demonstrated through extensive animal studies, wherein IF inhibited tumor growth by impeding glucose acquisition by the tumors for a short period [32].

Research proves that being overweight increases the risk of many cancers [33]. It has been demonstrated that high-fat diet- (HFD-) induced obesity impairs the CD8+ T cell function in mouse tumor microenvironment (TME) and accelerates tumor growth [34]. Recent studies have demonstrated that overweight and obesity are associated with higher risks of adenocarcinoma of the esophagus, gastric cardia, thyroid, pancreas, colon, rectum, endometrium, prostate, gallbladder, ovary, and breast, in addition to multiple myeloma [35].

Tumor cell growth depends on high levels of glucose for energy, amino acids for nitrogen, and synthetic precursors for proliferation; moreover, excessive reduction in circulating glucose and amino acids during IF can effectively inhibit cancer cell growth and provide a favorable environment for normal cells [36]. IGF-1 levels are positively correlated with carcinogenic risks, and deficiency of the growth hormone receptor also leads to IGF-1 deficiency, which reduces the risk of cancer and DNA damage [37]. IF can reduce cancer risk and DNA damage by increasing the levels of insulin-like growth factor inhibitory protein and ketone bodies as well as reducing IGF-1, insulin, and glucose concentrations [38]. The long-term success of antitumor therapy is mainly dependent on the patient's ability to restore anticancer immune monitoring, and IF can improve the efficacy of anticancer chemotherapy by inducing autophagy in malignant cells as well as anticancer immune responses [39].

AMP-activated protein kinase (AMPK) is one of the central regulators of metabolism in eukaryotic cells and organisms. AMPK is activated when intracellular ATP production is reduced [40]. In the presence of nutrient deficiency, it can act as a metabolic checkpoint to inhibit cell growth. While the most comprehensive mechanism by which AMPK regulates cell growth is through the inhibition of the mTOR pathway, in addition to regulating cell growth, mTORC1 also controls autophagy [40]. Furthermore, the oncogene chromosome 10 homologous loss of phosphatase gene, tensin, and tuberous sclerosis complex can inhibit the mTOR protein expression [41]. Under conditions of amino acid deficiency in protein synthesis, mTORC1 cannot cause phosphorylate dysregulation 51-like kinase 1 and autophagy-related gene 13 [42], thereby triggering autophagy and limiting the inflammatory response and inhibiting malignant cell development and progression [43].

In mouse models, IF improved the efficacy of chemotherapy regimens used for breast, melanoma, neuroblastoma, pancreatic, and colorectal cancers and reduced the harm caused by conventional therapies in humans [44].

**Figure 2:**
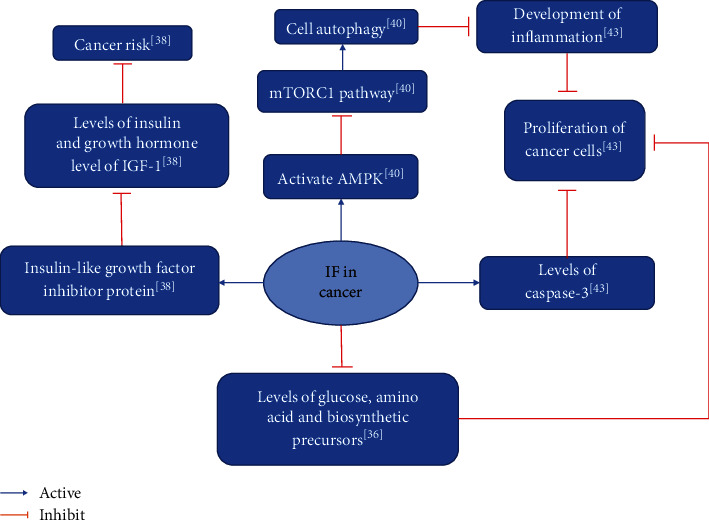
Summary of biological mechanisms of IF intervention in cancer.

Cooper et al. demonstrated that fasting for two days a week and splitting the calorie intake for the remaining five days effectively reduce IGF-1 levels and improve survival in a tumor model compared to other frequencies of IF [45]. De Groot Stefanie et al. demonstrated that FMD entails a significant benefit in enhancing the sensitivity of breast cancer cells to chemotherapy [46]. The experiments by Cui et al. demonstrated that IF reduces tumorigenesis, inhibits tumor formation, reduces the extent of liver damage during tumor formation, and maintains normal lipid metabolism [47] (Table 4).  Figure 2 shows the summary diagram of the biological mechanisms of simultaneous IF intervention in cancer.

### 2.4. IF in Neurological Diseases

Traumatic brain injury (TBI) is one of the most common causes of neurological injury in young people [48]. In animal models of stroke and Parkinson's disease, IF can avoid neuronal dysfunction and degeneration, induce beneficial cellular stress responses, stimulate the expression of genes encoding stress resistance proteins, and increase various neurodevelopmental factor production [49, 50]. Additionally, IF effectively increases histone deacetylase 1 (HDAC1) levels, which plays a neuroprotective role [51]. IF is also the least expensive and least risky way of promoting synaptic plasticity, increasing positive adaptations in the central nervous system, improving treatment outcomes, and even reversing pain [52].

IF improves LPS-induced memory deficit and increases levels of interleukin-10 (IL-10) in the hippocampus, inhibiting the development of chronic neurodegenerative diseases. IF exerts its anti-inflammatory effects by blocking LPS-induced increases in the levels of interleukin-1*α* (interleukin-10, IL-1*α*), interleukin-1*β* (interleukin-1*β*, IL-1*β*), tumor necrosis factor (TNF-*α*), and regulated upon activation normal T cell expressed and secreted (RANTES). While preventing a decrease in the hippocampal brain-derived neurotrophic factor levels, RANTES exerts anti-inflammatory effects [53]. IF increases the BDNF/CREB signaling pathway in the adult hippocampal brain, putting the brain into a neuroprotective state to resist injury and disease; the Notch 1 signaling pathway cooperates with the BDNF/CREB signaling pathway to allow the stem cells to differentiate the mature neurons while mediating the upregulation of the transcription factor Hes5-induced nestin [54]. However, the long-term safety of IF is often questioned [55].

Neurodegenerative diseases have become a pressing challenge for an aging population, with Alzheimer's disease (AD), Parkinson's disease (PD), Huntington's disease (HD), and others being the most widely studied [56]. The astrocyte caudal aquaporin-4 (AQP4) water channel-based glymphatic system is a pathway for the excretion of metabolic waste in the central nervous system (CNS). In the CNS, the alpha-1-syntropin (SNTA1) directly or indirectly anchors the AQP4 to the vascular-facing astrocyte membrane, and deletion of the SNTA1 reverses the polar distribution of the AQP4 to the vasculature [57]. At the same time, the AQP4 is involved in the perivascular clearance of A*β*. In AD patients, the localization of the astroglial AQP4 to the perivascular peduncle telangiectasias is reduced, while the localization to fine synapses is increased. The loss of perivascular AQP4 localization is associated with an increased pathological burden of local A*β* and tau proteins as well as a decline in cognition and function early in the disease process [58]. IF can improve the loss of AQP4 polarity in the cerebral cortex of AD model rats by reducing the AQP4-M 1/M23 and increasing the SNTA1 expression levels, thus effectively preventing AD [59]. PD is a neurodegenerative disease that entails motor dysfunction and loss of dopamine (DA) in the striatum. ADF has a protective effect on 1-methyl-4-phenyl-1,2,3,6-tetrahydropyridine- (MPTP-) induced subacute PD in mice. The intestinal barrier of mice was protected by MPTP in a study, which also inhibited the decrease of tyrosine hydroxylase (TH) and DA in the striatum [60].

**Figure 3:**
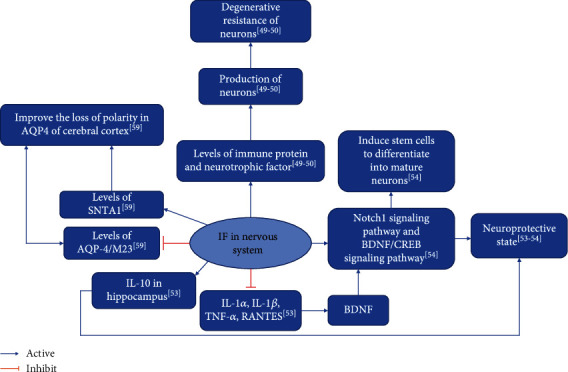
Summary of biological mechanisms of IF intervention in neurological diseases.

Experiments by Zhang et al. demonstrated that IF can effectively prevent AD [59]. Experiments by Zhang et al. demonstrated that ADF has neuroprotective effects and improves the composition of intestinal flora in PD mice and corrects abnormal changes in its metabolite short-chain fatty acids [60]. Experiments by Rubovitch et al. demonstrated that IF improves cognitive deficits in mTBI mouse models [61] (Table 5).  Figure 3 shows the summary diagram of the biological mechanisms of IF intervention in neurological diseases.

### 2.5. IF in Aging

Human life expectancy has nearly doubled in developed countries over the past century; however, this increase has also led to an increase in the prevalence of age-related diseases [62], such as neurodegenerative diseases, cardiovascular diseases, diabetes, osteoarthritis, and cancer [63]. Studies showed that IF increases the average lifespan of rats by 14-45% and mice by only 4-27% [64]. Further, DR increases fatty acid oxidation by maintaining mitochondrial network homeostasis and functional coordination with the peroxisome, thereby promoting longevity [65].

Clinical studies demonstrated that long-term IF improves cognitive disorders and reduces oxidative stress in middle-aged adults [66]. It also delays the onset of age-related brain damage [67]. Moreover, nutrient-sensing signaling pathways such as the AMPK, SIRT1, mTOR, and insulin/IGF-1 pathways are downregulated during IF, blocking cell proliferation and activating stress factors, thereby negatively regulating various aging signals [68]. IF can also protect the heart from ischemic damage [69], reduce body mass index and blood lipids [70], improve glucose tolerance [71], and reduce the incidence of coronary artery disease [72] by increasing levels of the growth hormone. This in turn increases lipolysis and insulin secretion in addition to reducing other glucose metabolism pathway markers.

**Figure 4:**
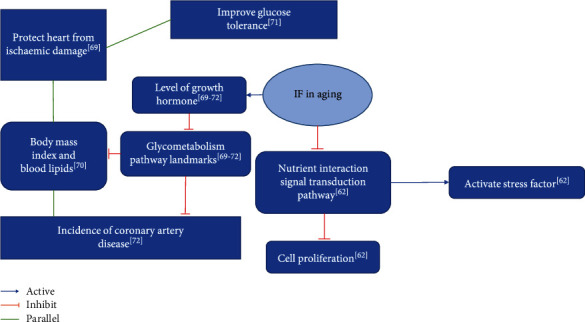
Summary of biological mechanisms of IF intervention in aging.

Humaira et al. demonstrated that TRF improves cardiometabolic health, alters the circadian rhythm, and has antiaging effects [73]. Yung et al. demonstrated that long-term IF alters intestinal flora, improves blood lipid levels, and controls body mass gain in preage obese rats [74]. Hu et al. demonstrated that IF slows the process of atherosclerosis development [75] (Table 6).  Figure 4 shows the summary diagram of the biological mechanisms of simultaneous IF intervention in aging.

### 2.6. IF in Biological Clock and Metabolism

The extensive role of the molecular and biological clocks in regulating nutritional and energy homeostasis and the biological circadian rhythm is coordinated by behavioral rhythms like activity-rest cycles and feeding-fasting cycles; these temporally coordinate a range of physiological processes to optimize metabolism [76]. At the same time, nutrient-sensing pathways can influence the biological clock, which can therefore be affected by changes in the timing of feeding [77, 78]. Animal studies demonstrated that the circadian resetting effect of IF can counteract the dysregulation of biological rhythms caused by a 24-hour fast [79–82]. Studies showed that Ramadan fasting is associated with evening hypercortisolism, while disturbances in the circadian rhythm mediate lower and disturbed hsCRP, albumin and liver enzyme levels, and altered adipokine patterns, adversely affecting cardiometabolism [83], without altering the energy expenditure. It increases fat oxidation and decreases carbohydrate oxidative metabolism without altering the energy expenditure [84, 85], and the effects may vary across countries due to different Ramadan practices. Additionally, IF has demonstrated its feasibility as a nonpharmacological means of preventing obesity by reshaping the intestinal flora and restructuring the circadian rhythm [86].

Adipose tissues are an important metabolically relevant part of the body, and adipokines released by adipocytes affect several physiological processes like insulin activity, lipid oxidation, glucose metabolism, and angiogenesis and remodeling [87, 88] fatty acid levels. These then stimulate the release of several proinflammatory peptides, thereby promoting insulin resistance and diabetes as well as the development of cancer [86–91]. IF leads to the depletion of hepatic glycogen stores and lipolysis of free fatty acids, which are metabolized in the liver to produce ketones, acetone, acetoacetate, and *β*-hydroxybutyric acid, which are transported to the brain [54].

Polycystic ovary syndrome (PCOS) is a common disorder of the endocrine system in women of reproductive age. Epidemiological studies showed that in some cases, women with PCOS exhibit infertility or low fertility as well as other metabolic alterations, such as insulin resistance, dyslipidemia, hyperinsulinemia, and obesity [92]. A study by Shafiee et al. found that the expressions of IGF-1, insulin-like growth factor-binding protein 1 (IGFBP1), and phosphatase and tensin homolog (PTEN) genes were significantly upregulated in the endometrium of women with PCOS and endometrial cancer (EC) compared to their study controls. The upregulation of IGF-1, IGFBP1, and PTEN was independent of systemic estradiol and androgen levels as well as estradiol levels BMI, HOMA-IR, and waist-hip-ratio (WHR) [93]. Considering the extreme importance of insulin receptors and compensatory hyperinsulinemia, the hypothesis that different fasting regimens reduce IGF-1, IGFBP1, glucose, and insulin levels and thus benefit ovarian function, androgen excess, and infertility in women with PCOS was proposed [94].

Experiments by Deng et al. demonstrated that long-term IF effectively improves inflammation levels [15]. Froy et al. demonstrated that IF can affect the circadian rhythm depending on the timing of food availability [82] (Table 7).

### 2.7. Summarizing the Biological Mechanisms of IF

**Figure 5:**
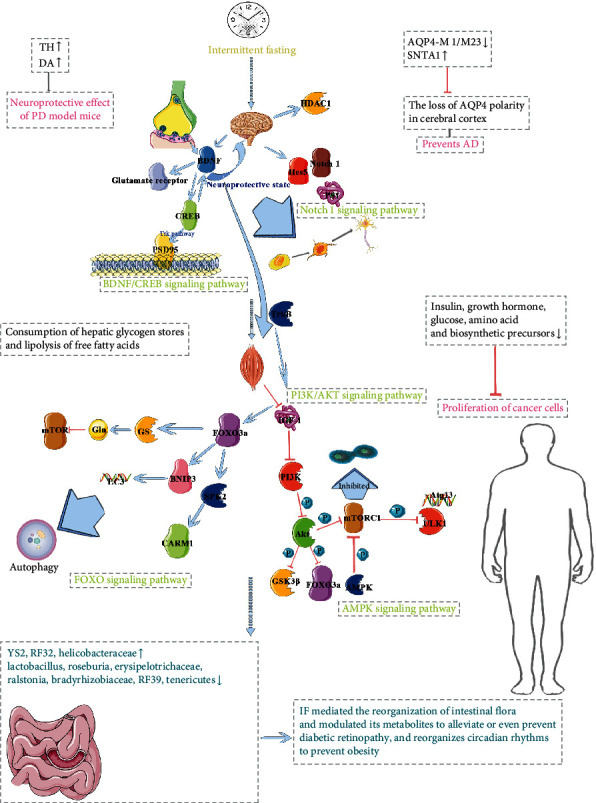
Summary of multiple biological mechanisms of IF. ULK1: uncoordinated 51-like kinase-1; mTORC1: mammalian target of rapamycin protein complex-1; BDNF: brain-derived neurotrophic factor; CREB: cAMP-response element-binding protein; IGF-1: insulin-like growth factor-1; AMPK: AMP-dependent protein kinase; PS-1: presenilin-1; PSD95: postsynaptic density-95; TrkB: tropomyosin receptor kinase B; Atg13: autophagy-associated gene 13; BNIP3: Bcl2/adenovirus E1B 19 kD-interacting protein 3; SPK2: sphingosine kinase-2; CARM1: coactivator-associated arginine methyltransferase-1; GS: glutamine synthetase; Gln: glutamine; PI3K: phosphatidylinositol-3-kinase; Akt: protein kinase B; GSK3*β*: glycogen synthase kinase 3*β*.

IF mediates the Notch1 and BDNF/CREB signaling pathways to induce stem cell differentiation to mature neurons [54] and improve diabetes-induced cognitive impairment [25]. It induces cellular autophagy through the PI3K/AKT, FOXO, and AMPK signaling pathways to promote myocyte renewal [95], prevent cancer cell proliferation and spread [41–43], and delay aging [68]. IF also mediates the reorganization of intestinal flora and modulates its metabolites to alleviate or even prevent diabetic retinopathy [27]. It also reorganizes the circadian rhythm to prevent obesity [86].  Figure 5 shows the summary diagram of the multiple biological mechanisms of IF at the same time.

## 3. The Negative Effects and Limitations of IF

IF is a convenient and simple diet with many health-promoting benefits, but it also has several limitations.

### 3.1. The Negative Effects and Limitations of IF in Cardiovascular Diseases

ADF reduces the cardiac reserve function and diastolic insufficiency in rats, leading to smaller cardiomyocytes and increased myocardial fibrosis, with fibrotic hearts exhibiting diastolic dysfunction at rest and reduced diastolic and systolic reserve capacity [96]. Acute fasting reduces tolerance to central hypovolemia by blunting the increase in peripheral vascular resistance; it may also lead to premature loss of compensation compared to the normal feeding state [97].

### 3.2. The Negative Effects and Limitations of IF in the Endocrine System

In young mice with inadequate glucose metabolism, IF leads to several unfavorable changes, including elevated islet cell apoptosis and reactive oxygen species (ROS) production, reduced islet tissue volume, and a substantial increase in insulin secretion [98]. Diabetic patients during IF exhibit hypoglycemia, ketoacidosis, dehydration, hypotension, and thrombosis [99, 100]. Contrastingly, in pregeriatric obese rats, long-term IF alters intestinal flora, improves lipid levels, and controls body mass gain; it also increases blood glucose levels and reduces glucose tolerance [74].

### 3.3. The Negative Effects and Limitations of IF in the Reproductive System

The effect of IF on female fertility manifests in the form of anorexia nervosa, which develops into nutritional infertility, due to inadequate nutritional intake [101]. The significant effects of IF-DR regimens on body weight, blood glucose, estrous cycle, and multiple hormone levels in young rats adversely affect the entire hypothalamic-pituitary-gonadal axis, which in turn affects reproduction in young rats [55].

IF dietary restriction negatively affects reproduction in young rats of the Wistar strain because of its adverse effects on the intact hypothalamic-pituitary-gonadal axis, and it may explain the underlying mechanisms to understand the clinical basis of nutritional infertility [55].

### 3.4. The Negative Effects and Limitations of IF in Other Systems

IF promotes a greater accumulation of triacylglycerides in white adipose tissues by increasing the expression of lipid storage-related genes, such as adipose-specific protein 27 [102]. Another study demonstrated that IF weekly twice did not enhance the survival of mice in a small model of tumor [103].

Intermittent access extensively promotes weight gain, fasting hyperglycemia, and psychomotor arousal during early withdrawal in male rats. Stricter access promotes greater binge-style intake and fat accumulation, while longer access promotes greater evidence of food reward tolerance [104].

Elderly people training at least thrice a week during Ramadan may improve cognition but cause impaired sleep quality [105].

Repeat sprint ability (RSA) is now recognized as an important fitness component in the performance of team sports. It is widely described as the ability to perform repeated short-distance (~3-4 s, 20-30 m) sprints, with only a short recovery (10-30 s) between sprints. IF reduces speed and power in the initial run of the RS in the second set (2 sets: 5 × 5 s maximal sprint, 25 s recovery in between, 3 min recovery between sets) due to reduced vertical stiffness [106].

### 3.5. Summarizing the Biological Mechanisms of IF in the Context of Negative Effects

**Figure 6:**
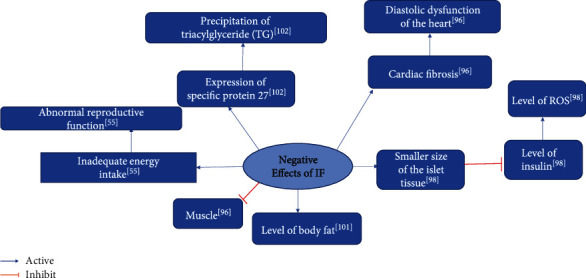
Summary of the negative effects of IF.

Experiments by Ahmet et al. demonstrated that chronic ADF causes diastolic dysfunction and reduced cardiac reserve in rats [96]. In the experiments by Sushil et al., IF adversely affected reproduction in young animals [55]. In the experiments by Munhoz et al., IF proved effective for weight loss, but its long-term safety has been questioned [98]. Park et al. demonstrated the unsuitability of IF for patients with glucose metabolism disorders [107]. Boujelbane et al. claimed that exercise is more beneficial for the elderly during Ramadan [105]. Cherif et al. claimed that three days of IF decrease RSA in athletes [106], and Sushil et al. indicated that IF adversely affects the reproductive system of mice [55] (Table 8).  Figure 6 shows the summary diagram of biological mechanisms of IF in the context of negative effects at the same time.

To summarize, IF has some negative effects and limitations for specific age and gender groups, but these need to be confirmed by more high-quality studies.

## 4. Summary and Prospects

IF entails a cyclical pattern of eating, with no or minimal energy intake for a period of time after normal eating. Therefore, IF also has biological effects. Its health-promoting mechanism has been studied through the mediation of multiple biological pathways. IF has a higher adherence rate than CR. It not only is an alternative to CR for reducing obesity symptoms, but it is also a nonpharmacological treatment that may extend life expectancy and improve the quality of life during old age. The effectiveness and safety of its health-promoting effects need to be systematically studied. Future research should emphasize improving its negative effects. The resultant improved approach should then be used as adjunctive therapy for chronic diseases like diabetes and cancer.

## Figures and Tables

**Table 1:** Classification of intermittent fasting.

English name	Abbreviation	Definition description
Alternate-day fasting	ADF	It consists of “eating days” (eating normally or consuming 125 to 150 percent of the calories required for a regular diet) and “fasting days” (fasting for 24 hours or limiting energy intake to 25 percent or less), which do not restrict overall calorie intake but only change the frequency of eating.
Time-restricted fasting	TRF	It allows energy intake freely within a controlled period (usually 3-12 h per day).
Modified fasting	MF	① ADF-25% (fasting day 25% CR); ② ADF-50% (fasting day 50% CR); ➂ ADF-100% (fasting day 100% CR)
Fasting-mimicking diet	FMD	Five-day monthly meal plan: limit 1.1 kcal calories on the first day and 0.75 kcal on the second to five days, and eat the rest of the time freely.

**Table 2:** Information about IF and obesity.

Information	Method	Sample feature	Results	Discussion
Bhutani [9]	Nonrandomized controlled experiments in humans	*n* = 64 Obese patients	BW, VF, TG, TC, LDL↓	ADF can effectively reduce body weight and improve the health of patients.
Ara [10]	Nonrandomized controlled experiments in humans	*n* = 60 Healthy men	BW, BMI, TC, LDL-C↓; HDL (NA); C↑↑	Ramadan can effectively reduce body weight and improve obesity-related health indicators.
Gur [11]	Randomized controlled experiments in humans	*n* = 78 Healthy pregnant women	HDL↑; LDL, TC, TG, BW (NA)	Ramadan can lead to a decrease in VFT without affecting fetal development or amniotic fluid levels in healthy pregnant women.
Madkour [13]	Nonrandomized controlled experiments in humans	*n* = 56 Obese patients	BW, BMI, FM, VFA, IGF-1, TC, TG, HDL, TBW, SBP, W↓↓; INS, IR, Glu (NA)	Ramadan has a short-term protective effect against oxidative stress in obese subjects.
John [14]	Randomized controlled experiments in humans	*n* = 100 Overweight and obese patients	VAT, FM, FFM, IR, SAT, IGF-1↓; Glu, LEP, TNF-*α*, IL-6↑	CR and ADF similarly improve the FFM : total mass ratio and reduce leptin after a 24-week intervention.
Deng [15]	Experiments in animals	*n* = 36 Obesity mice	BW, LEP, WAT, LPS, HDL, ADPN↓; INS, LOC↑	IF can alleviate the metabolic disorder of white adipose tissue in the condition of high-fat diet.

Note: NA: not analyzed; TG: triglyceride; TC: serum total cholesterol; LDL-C: low-density lipoprotein cholesterol; HDL-C: high-density lipoprotein cholesterol; INS: insulin; IR: insulin resistance; BW: weight; VAT: visceral adipose tissue; SAT: subcutaneous adipose tissue; FFM: free fat mass; FM: fat mass; VF: visceral fat content; WAT: white adipose tissue; LEP: leptin; ADPN: adiponectin; Glu: glucose; VFA: visceral fat area; VFT: visceral adipose tissue; HDL: high-density lipoprotein; LDL: low-density lipoprotein; TBW: total body water content; W: waist circumference; SBP: systolic blood pressure; LOC: liver organ coefficient; LPS: inflammation factor lipopolysaccharide.

**Table 3:** Information about IF and type 2 diabetes.

Information	Method	Sample feature	Results	Discussion
Turki [28]	Nonrandomized controlled experiments in humans	Patients with T2DM (*n* = 5) Healthy control (*n* = 7)	FM↓↓; MP, ghrelin, GIP, GLP-1, PYY, PP↑	Ramadan can reduce the body weight of diabetics effectively.
Arnason [29]	Randomized controlled experiments in humans	*n* = 10 Overweight patients with T2DM	Glu↓; IR, IM (NA)	ADF can effectively improve key indicators such as BW and Glu in patients with T2DM.
Klempel [30]	Nonrandomized controlled experiments in humans	*n* = 54 Prediabetes	BW, Glu, IR↓	MF can effectively prevent the occurrence of diabetes.

Note: NA: not analyzed; IR: insulin resistance; BW: body weight; FM: adiposity; MP: metabolic parameters; GIP: glucose-dependent insulin-releasing peptide; GLP-1: glucagon-like peptide-1; PYY: gastrointestinal peptide tyrosine; PP: pancreatic polypeptide; IM: inflammatory marker; Glu: blood glucose.

**Table 4:** Information about IF and cancer.

Information	Method	Sample feature	Results	Discussion
Cooper [45]	Nonrandomized controlled experiments on animals	*n* = 105 Male Fox Chase SCID mice injected in the flank with 1 × 10^5^ LAPC-4 cells were randomized to seven groups	IGF-1↓; survival percentage↑ (after 20 days)	Tumor growth was not significantly inhibited, but the survival rate was improved.
Stefanie [46]	Randomized controlled experiments in humans	*n* = 131 HER2-negative II/III breast cancer patients	Toxicity grade III/IV of the subjects (NA); DNA damage in T lymphocytes↓	FMD has significant benefits in increasing the sensitivity of breast cancer cells to chemotherapy.
Cui [47]	Nonrandomized controlled experiments on animals	IF group (*n* = 8) AL group (*n* = 8)	TFR, TV, damage to the liver caused by liver cancer↓	IF can reduce tumorigenesis, inhibit tumorigenesis, reduce the degree of liver injury during tumorigenesis, and maintain normal lipid metabolism.

Note: SCID: severe combined immunodeficiency; LAPC-4: Los Angeles prostate cancer-4; NA: not analyzed; TFR: tumor formation rate; TV: tumor volume; Glu: blood glucose.

**Table 5:** Information about IF and neurological diseases.

Information	Method	Sample feature	Results	Discussion
Zhang [59]	Nonrandomized controlled experiments on animals	*n* = 40 APPswe/PSIdE9 double transgenic mice	ADF improved AQP4 polarity	IF caused the loss of AQP4 polarity in the cerebral cortex of the target mice, inhibited the expression of AQP4 and AQP4-M1 and the increase of AQP4-M1/M23, and decreased the expression of SnTA1.
Zhang [60]	Nonrandomized controlled experiments on animals	C57BL/6 mice with acute PD randomly divided into four groups: NS+AL group, NS+ADF group, MPTP+AL group, MPTP+ADF group	ADF: motor dysfunction, DA, 5-HT, TH, *Prakk1*, *Tjp1* ↑; DOPAC, HVA, 5-HIAA↓	ADF has protective effects on intestinal barrier and nerves and maintains the integrity of intestinal epithelium in PD mice.
Rubovitch [61]	Nonrandomized controlled experiments on animals	ICR male mouse control group TBI group (AL group, IF group, CR group)	IF: prevented the significant decrease of preference index, SIRT1 in TBI mice	IF is effective in ameliorating cognitive deficits in a TBI model when initiated after the brain injury.

Note: AL: fed ad libitum; NS: normal saline; ADF: alternate-day fasting; MPTP: 1-methyl-4-phenyl-1, 2, 3, 6-tetrathydropyridine; transgenic mice were transferred to both the amyloid precursor protein gene (APP) K670N mutant gene and the early ageing protein 1 gene (PS1) E9 mutant gene. APPswe/Pside9 double transgenic mice; Prakk1: mRNA of AMPK; Tjp1: mRNA of ZO-1; DOPAC: 3,4-dihydroxyphenylacetic acid; HVA: homovanillic acid; 5-HIAA: 5-hydroxyindoleacetic acid; ICR: Institute of Cancer Research; TBI: traumatic brain injury.

**Table 6:** Information about IF and senescence.

Information	Method	Sample feature	Results	Discussion
Humaira [73]	Nonrandomized controlled experiments in humans	*n* = 11 Overweight adults	Glu↓; ketones, cholesterol, BDNF, SIRT1, LC3A↑	TRF improves cardiac metabolic health, alters circadian rhythms, and has antiaging effects.
Rong [74]	Nonrandomized controlled experiments on animals	*n* = 23 Male elderly Wistar rat tumorigenesis	BW, HDL-C, LDL-C↓↓; GT↓; FPG↑↑	IF can improve the intestinal flora and reduce the body mass and blood lipid levels of obese rats in the presenile period.
Hu [75]	Nonrandomized controlled experiments on animals	ApoE mice with atherosclerosis (*n* = 6) AS mouse IF group (*n* = 32) AL group (*n* = 32)	MMP-9, CC, FC, LC↓	IF reduced the area of atherosclerotic plaque and delayed the progression of atherosclerosis in AS mice.

Note: BW: body weight; HDL-C; LDL-C; Glu: blood glucose; FPG: fasting glucose; GT: glucose tolerance; CC: cholesterol crystals; FC foam cells; CF fibrous cap; LC lipid core; LC3A: microtubule-associated protein 1A/1B-light chain 3A.

**Table 7:** Information about IF and metabolism.

Information	Method	Sample feature	Results	Discussion
Deng [15]	Nonrandomized controlled experiments on animals	*n* = 36 Obesity model mice	LPS, *TNF-α*, *IL-1β*↓↓	The short-term improvement effect of IF is limited, while the long-term intermittent fasting can significantly improve the inflammatory state.
Froy [82]	Randomized controlled experiments on animals	FVB/N wild-type (W) mice and FVB/N mice-derived transgenic MUPA mice	*mPer2*, *mCry1*, *mClock*, *mBmal1*↓	IF can affect circadian rhythms depending on the timing of food availability and induces a metabolic state that affects the suprachiasmatic nucleus (SCN) clock.

Note: the FVB/NJ mouse strain has its origins in a colony of outbred Swiss mice established in 1935 at the National Institutes of Health. FVB/NJ mice; LPS: lipopolysaccharide; Mper2: mammalian period; Mcry1: mammalian cryptochrome 1; Mclock: mammalian clock proteins; Mbmal1: mammalian brain–muscle-Arnt-like 1.

**Table 8:** Information on negative effects of IF.

Information	Method	Sample feature	Results	Discussion
Ahmet [96]	Nonrandomized controlled experiments on animals	ADF group (*n* = 20) AL group (*n* = 20)	Cardiomyocyte size, diastolic and systolic reserve capacity↓; myocardial fibrosis↑	Chronic ADF in rats leads to the development of diastolic dysfunction and reduced cardiac reserve.
Sushil [55]	Nonrandomized controlled experiments on animals	Wistar strain young adult male and virgin cycling female albino rats divided into the control group and the IF-DR group	Estrous cycle disrupted, ovarian weight, testosterone↓; Glu, LEP, LH↓↓; estradiol↑↑	IF-DR has a negative effect on the reproductive function of female rats and disrupts the estrus cycle of female rats.
Munhoz [98]	Nonrandomized controlled experiments on animals	Wistar rats randomly divided into the control group and the ADF group	LG, BW, IA↓; length of the tibia↓↓; Lee's index, FM↑; P-Akt (NA)	IF is effective for weight loss, but its long-term safety has been questioned.
Park [107]	Nonrandomized controlled experiments on animals	Sprague-Dawley rats randomly divided into four groups: HP-AL group, HF-AL group, HP-IMF group, HF-IMF group	GT, IR, dyslipidemia↑	Patients with glucose metabolism disorder should not apply this method.
Cherif [106]	Nonrandomized controlled experiments in humans	Males (*n* = 21) CS and FS (7 days in between)	3d-IF impaired speed and power through a decrease in vertical stiffness during the initial runs of the second set	3d-IF impaired speed and power through a decrease in vertical stiffness during the initial runs of the second set, 3d-IF improved HDL-C and TG profiles while maintaining TC and LDL-C levels.
Boujelbane [105]	Nonrandomized controlled experiments in humans	Sedentary group (*n* = 32) Physically active group (*n* = 26)	Physically active group: executive function, attention, inhibition, associative memory, and recognition memory↑↑	Elderly people who continue to train at least three times a week during Ramadan may improve their cognitive performance, although sleep quality is impaired.

## Data Availability

The data supporting this systematic review are from previously reported studies and datasets, which have been cited. The processed data are available from the corresponding authors upon request.
